# T-Cell Receptor and Immune Gene Expression Pharmacodynamics for Durvalumab Alone and with Tremelimumab or Bevacizumab in Unresectable Hepatocellular Carcinoma

**DOI:** 10.1158/1078-0432.CCR-25-1592

**Published:** 2025-12-08

**Authors:** Robin K. Kelley, Young Lee, James Conway, John F. Kurland, Patricia McCoon

**Affiliations:** 1 Helen Diller Family Comprehensive Cancer Center, University of California, San Francisco, California.; 2 Oncology R&D, AstraZeneca, Gaithersburg, Maryland.; 3 Oncology R&D, AstraZeneca, Waltham, Massachusetts.

## Abstract

**Purpose:**

In the phase I/II Study 22 (NCT02519348) trial, objective response rates were 24.0% with STRIDE (single tremelimumab regular-interval durvalumab), 21.3% with durvalumab plus bevacizumab (D + B), and 11.5% with durvalumab monotherapy in unresectable hepatocellular carcinoma (uHCC). Increased proliferating CD8^+^ T cells were associated with improved efficacy of STRIDE versus durvalumab monotherapy. Here, analyses of changes in T-cell clonal expansion and gene expression signatures (GES) in peripheral blood were performed to explore the mechanisms of action associated with the anticancer activity of STRIDE and D + B versus durvalumab monotherapy.

**Patients and Methods:**

Participants with uHCC and no prior immune checkpoint inhibitor therapy were enrolled. DNA and RNA were isolated from peripheral blood collected at baseline and at the end of the first treatment cycle. Baseline values and changes from baseline in T-cell clonality and GES were measured across treatment arms, and associations with radiographic response were assessed.

**Results:**

There were no significant differences in baseline richness or Simpson clonality of T cells across treatment arms. STRIDE, but not D + B, elicited an increase in the number of expanded T-cell clones versus durvalumab monotherapy; the increase was associated with clinical response. Both STRIDE and D + B upregulated IFNγ response GES compared with durvalumab alone, but other immune-related changes differed, with STRIDE showing upregulation of CD4^+^ and T effector signatures, whereas D + B upregulated IFNα response and both myeloid cell and endothelial GES.

**Conclusions:**

These findings suggest that STRIDE and D + B have distinct, and potentially complementary, mechanisms of action in uHCC.

**Translational Relevance**

STRIDE (single tremelimumab regular-interval durvalumab) showed promising and higher clinical activity [objective response rate (ORR), 24.0%] versus durvalumab monotherapy (ORR, 11.5%) in participants with unresectable hepatocellular carcinoma (uHCC) who had disease progression on, were intolerant to, or refused sorafenib in Study 22 (NCT02519348). In the same study, the ORR with durvalumab plus bevacizumab in treatment-naïve participants with uHCC was 21.3%. This exploratory analysis of peripheral blood samples showed that STRIDE, but not durvalumab plus bevacizumab, is associated with expansion of new and existing T-cell clones versus durvalumab monotherapy, whereas durvalumab plus bevacizumab demonstrated a decrease in the fraction of productive T cells. In addition, tremelimumab and bevacizumab elicit distinct gene expression changes when combined with durvalumab. These findings suggest the intriguing possibility that an even greater benefit in uHCC is attainable if dual checkpoint inhibition and VEGF inhibition can be accomplished safely.

## Introduction

Immune checkpoint inhibitor (ICI) regimens are standard-of-care first-line therapy for unresectable hepatocellular carcinoma (uHCC; refs. 1–4). Combination treatment with two ICIs, an anti–PD-(L)1 plus anti–cytotoxic T-lymphocyte–associated protein 4 (anti–CTLA-4), or an ICI plus anti-VEGF in the first-line setting has demonstrated significant improvements in overall survival (OS) versus the former standard of care, sorafenib, in phase III clinical trials of participants with uHCC (1, 2, 5). Although ICI monotherapies have shown clinical activity in participants with uHCC with numerical improvements in objective response rate (ORR), duration of response (DoR), and OS, including nivolumab (anti–PD-1), durvalumab (anti–PD-L1), and tislelizumab (anti–PD-1), collectively, these agents did not show superiority for OS versus sorafenib (1, 6, 7). Taken together, these data indicate that ICI combinations seem critical to delivering meaningful effects in uHCC.

Study 22 (NCT02519348) was a phase I/II trial that consisted of four parts and assessed immunotherapy-based regimens in 379 participants with uHCC (8, 9). Results from parts 2 and 3 of Study 22 have been previously published (8) and showed that the STRIDE regimen (a single priming dose of tremelimumab plus durvalumab at cycle 1, followed by durvalumab monotherapy every 4 weeks) had the highest ORR (24.0%) compared with durvalumab monotherapy (11.5%), tremelimumab monotherapy (7.2%), or tremelimumab 75 mg plus durvalumab (T75 + D; 9.5%). The longest median OS was also observed with STRIDE (18.73 months) compared with durvalumab monotherapy (13.57 months), tremelimumab monotherapy (15.11 months), or T75 + D (11.30 months; ref. 8). Additional findings from an exposure–response analysis of STRIDE and durvalumab support that STRIDE combination treatment is necessary and sufficient to drive better outcomes than durvalumab alone in participants with uHCC (10).

In part 4 of Study 22, participants who had not received prior systemic therapy were allocated to receive durvalumab plus bevacizumab (9). Results from part 4 showed promising clinical activity for durvalumab plus bevacizumab in the first-line setting, with an ORR of 21.3% (9). The median duration of follow-up was shorter in part 4 (13.73 months) compared with parts 1 to 3 and resulted in the median OS not being reached; however, the 12-month OS rate was 71.8% (9). These OS data are consistent with the outcomes reported from treatment with atezolizumab plus bevacizumab (2) and sintilimab plus a bevacizumab biosimilar (11), supporting a class effect for the efficacy of this combination strategy.

The mechanisms underpinning the antitumor activity of ICIs may vary depending on whether they are used as monotherapy (e.g., PD-L1 alone) or in combination with other anticancer therapies, such as VEGF inhibitors or anti–CTLA-4 agents. Flow cytometry analyses from parts 2 and 3 of Study 22 on peripheral blood samples collected on day 15 showed differences in the number of proliferating T cells across treatment arms (STRIDE, durvalumab monotherapy, tremelimumab monotherapy, and T75 + D), with an increased number of proliferating T cells in the STRIDE arm compared with the durvalumab monotherapy arm; the expansion of proliferating CD8^+^ lymphocytes was associated with objective responses (8). These data suggest that differences in proliferating T cells may be due to exposure to CTLA-4 (tremelimumab) and whether they are used as monotherapy or in combination with durvalumab.

Although biomarker results from part 4 of Study 22 have not previously been reported, the combination of ICI with an antibody targeting VEGF is anticipated to lead to antitumor effects by normalizing vasculature in the tumor, increasing the maturation of dendritic cells, resulting in improved antigen presentation, and promoting tumor infiltration of CD8^+^ T cells (12). A previous integrated molecular analysis of baseline tumor samples from participants with HCC treated with atezolizumab plus bevacizumab showed high expression of baseline PD-L1 mRNA and a T-effector gene expression signature, enrichment of inflammation response pathways, and high density of CD8^+^ T cells in the tumor microenvironment, which were associated with improved outcomes (13).

The Study 22 trial design presented a unique opportunity to compare the pharmacodynamic changes that accompany dual-ICI therapy versus ICI plus VEGF inhibitor combinations and how these changes may contribute mechanistically to the improved efficacy observed for ICI combinations versus ICI monotherapy. Here, we aim to elucidate commonalities and differences in mechanisms of action associated with STRIDE and durvalumab plus bevacizumab compared with durvalumab monotherapy in Study 22. Specifically, we conducted exploratory analyses to evaluate peripheral blood samples for changes in T-cell clones and gene expression signatures and their associations with clinical response.

## Patients and Methods

### Study design and participants

Study 22 (NCT02519348) was an open-label, multicenter, international, phase I/II trial conducted in four parts that assessed the safety, tolerability, and clinical activity of tremelimumab and durvalumab as monotherapy or combination therapy in participants with uHCC at the data cutoff of November 06, 2020 (8). Eligibility criteria and study methodology have been previously described (8). Briefly, eligible participants were aged ≥18 years (≥20 years in Japan) with histologically confirmed uHCC (8). In parts 1 to 3 of Study 22, participants had disease progression on, were intolerant to, or refused first-line sorafenib (8). Part 1 was a safety run-in and efficacy gating of T75 + D (tremelimumab 75 mg plus durvalumab 1,500 mg once every 4 weeks for four dosages, followed by durvalumab monotherapy once every 4 weeks). In part 2a, participants were randomized to receive either durvalumab monotherapy (durvalumab 1,500 mg once every 4 weeks), tremelimumab monotherapy (tremelimumab 750 mg once every 4 weeks for a total of seven dosages and then once every 12 weeks), or the T75 + D regimen. In part 2b, participants were allocated to receive the STRIDE regimen consisting of a single dose of tremelimumab 300 mg with durvalumab 1,500 mg, followed by durvalumab 1,500 mg once every 4 weeks. In part 3, participants were randomized to receive either durvalumab monotherapy, tremelimumab monotherapy, the T75 + D regimen, or the STRIDE regimen. Enrollment in the T75 + D arm in part 3 was closed after 45 participants were recruited following a protocol amendment. In part 4, participants who had not received prior systemic therapy were allocated to receive durvalumab plus bevacizumab (durvalumab 1,120 mg plus bevacizumab 15 mg/kg once every 3 weeks; ref. 9).

Study 22 was conducted in accordance with the Declaration of Helsinki and was consistent with the International Council for Harmonization and Good Clinical Practice guidelines, applicable regulatory requirements, and the AstraZeneca Bioethics Policy. Written informed consent was obtained from all participants before participation.

The primary objective of Study 22 was to assess safety. The key secondary objectives included efficacy endpoints of OS, ORR, and DoR. ORR and DoR were assessed by Blinded Independent Central Review (BICR) according to RECIST version 1.1 (8). A prespecified exploratory objective of Study 22 was to assess biomarker association with clinical response. Samples from parts 2, 3, and 4 of Study 22 were used to assess peripheral blood T-cell receptors and gene expression signatures.

### T-cell clone analysis and gene expression signature analysis

This analysis was conducted on whole-blood samples of participants collected at baseline and at the end of cycle 1 in PAXgene RNA tubes and stored frozen at −80°C (−112°F). The end of cycle 1 was on day 29 for the durvalumab and tremelimumab arms and day 22 for the durvalumab plus bevacizumab arm. Samples were split so DNA and RNA could be isolated from the same sample.

The quality and quantity of total RNA were assessed using an Agilent BioAnalyzer (Agilent Technologies, RRID: SCR_018043) and NanoDrop (Thermo Fisher Scientific, RRID: SCR_018042). To reduce the abundance of globin mRNA commonly present in blood samples, globin RNA was removed using the GLOBINclear-Human Kit (Thermo Fisher Scientific) according to the manufacturer’s protocol. Residual rRNA was further depleted from total RNA samples by hybrid capture with biotinylated probes. The rRNA-depleted RNA was fragmented and converted into indexed cDNA libraries using the TruSeq Stranded Total RNA Gold kit with Ribo-Zero (Illumina). The resulting cDNA libraries were quantified by qPCR, assessed for quality with an Agilent TapeStation (Agilent Technologies, RRID: SCR_018435), normalized, and sequenced as 100 bp paired-end reads on an Illumina sequencing platform (Illumina, RRID: SCR_016387).

Complementarity-determining region 3 (CDR3) sequencing of T-cell receptor β (TCR-β) using the immunoSEQ assay (Adaptive Biotechnologies) and RNA sequencing (RNA-seq; Q Squared Solutions) was performed. Genomic DNA extracted from whole blood was submitted to Adaptive Biotechnologies for TCR-β repertoire analysis. Sequencing of CDR3 of human TCR-β chains was performed using Adaptive Immunosequencing (Adaptive Biotechnologies). Extracted genomic DNA was amplified in a bias-controlled multiplex PCR, followed by high-throughput sequencing. Sequences were collapsed and filtered to identify and quantitate the absolute abundance of each unique TCR-β CDR3 region for further analysis as previously described (14–16).

Changes in T-cell clonality and gene expression signatures between baseline and end of cycle 1 were analyzed. These analyses were performed in the respective biomarker-evaluable populations, which included any participant in the intention-to-treat population with PAXgene samples that were evaluable by either immunoSEQ assay (immunoSEQ-evaluable participants) or RNA-seq (RNA-seq–evaluable participants) at both baseline and on treatment at the end of cycle 1. Gene signatures described in Bagaev and colleagues (Bagaev immune set), Jiménez-Sánchez and colleagues (Consensus tumor microenvironment), and Liberzon and colleagues (Hallmark) were evaluated for changes at the end of cycle 1 versus baseline (17–19). For the gene expression analysis, log_2_ transcripts per million expression values were analyzed using the “GSVA” (version 1.50.5; RRID: SCR_021058) package of R (version 4.3.1) software to assess single-sample gene set enrichment analysis (RRID: SCR_003199) signature scores. To analyze signature statistics over time, a mixed-effects model was applied using the “lme4” (version 1.1–35.5; RRID: SCR_015654) package in R to account for random participant effects.

Additional exploratory analysis of expanded T-cell clones was conducted to assess for associations with clinical response across all treatment arms and the pooled tremelimumab-containing arms at day 29. Statistical measures of biomarker data from the biomarker-evaluable populations, included for each figure as part of the figure legend, were descriptive only and considered exploratory.

### Statistical analysis of TCR-β sequencing results

Two quantitative components of diversity were compared across samples in this study. First, Simpson clonality was calculated on productive rearrangements by the formula

$$\sqrt{\overset{R}{\underset{i=1}{\sum }}p_{i}^{2}}$$

in which *R* is the total number of productive rearrangements and $p_{i}$ is the productive frequency of rearrangement i. Values of Simpson clonality range from 0 to 1 and measure how evenly receptor sequences (rearrangements) are distributed. Clonality values approaching 0 indicate an even distribution of frequencies, whereas values approaching 1 indicate an increasingly asymmetric distribution in which one to a few clones are present at high frequencies.

Sample richness was calculated as the number of unique productive rearrangements in a sample after computationally downsampling to a common number of templates to control for variation in sample depth or T-cell fraction. Repertoires were randomly sampled without replacement five times, and the mean of these values is reported as downsampled rearrangements.

T-cell fraction was calculated by taking the total number of T-cell templates detected and dividing by the total number of nucleated cells. The total number of nucleated cells was determined using a panel of reference genes as part of immunosequencing.

Clonal expansion/contraction was calculated according to a binomial distribution framework as described previously (20). In brief, for each unique rearrangement, we considered the combined template count between the two samples being compared as a fixed number of “trials” in a two-sided binomial test. We calculated the probability of the observed template counts in each sample under the null hypothesis that these templates were evenly distributed between the two samples relative to their respective repertoire sizes (i.e., total productive templates). Clones that were more unequally distributed relative to this expected proportion would result in a lower binomial probability. The Benjamini–Hochberg procedure was used to control the FDR at 0.01 (21). All statistical analyses were performed in R (version 4.1, RRID: SCR_001905).

## Results

### Study population

In parts 2, 3, and 4 of Study 22, a total of 379 participants were included in the durvalumab monotherapy (*n* = 104), durvalumab plus bevacizumab (*n* = 47), tremelimumab monotherapy (*n* = 69), T75 + D (*n* = 84), and STRIDE (*n* = 75) arms, representing the clinically evaluable population. The demographic and baseline characteristics of participants were generally balanced across treatment arms, regardless of exposure to prior therapy, except for first-line therapy which was expected as participants in parts 2 and 3 had disease progression on, were intolerant to, or refused first-line sorafenib, whereas those in the part 4 durvalumab plus bevacizumab combination arm had received no prior systemic therapy for HCC (Supplementary Tables S1–S3; refs. 8, 9). ImmunoSEQ-evaluable [139 of 379 (36.7%)] and RNA-seq–evaluable [159 of 379 (42.0%)] paired samples were available from a subset of participants (Table 1).

**Table 1.** Overview of response or T-cell clonality in clinically evaluable participants, RNA-seq–evaluable participants, and immunoSEQ-evaluable participants.

​	Durvalumab plus bevacizumab^a^	Durvalumab monotherapy^b^	T75 + D^b^	STRIDE^b^	Tremelimumab monotherapy^b^
Clinically evaluable participants	​	​	​	​	​
Participants, *n*	47	104	84	75	69
Median duration of follow-up in censored participants, months^c^ (25th, 75th percentile)	13.73 (4.86, 18.00)	23.18 (1.84, 44.29)	29.82 (0.03, 43.14)	24.61 (0.95, 35.58)	31.03 (1.81, 44.02)
					
ORR, %	21.3	11.5	9.5	24.0	7.2
Median DoR, months^d^^,^^e^ (25th, 75th percentile)	NR (NC, NC)	14.95 (8.54, NR)	13.21 (10.15, NR)	18.43 (5.59, 23.95)	23.95 (4.07, NR)
					
RNA-seq–evaluable participants	​	​	​	​	​
Participants with samples, *n*	37	35	28	38	21
ORR, %	18.9	14.3	3.6	26.3	4.8
immunoSEQ-evaluable participants	​	​	​	​	​
Participants with samples, *n*	37	31	26	28	17
ORR, %	29.7	16.1	7.7	32.1	5.9
Median expanded T-cell clones at the end of cycle 1 (25th, 75th percentile)	18 (5, 37)	32 (13, 49)	36 (15, 77)	56 (33, 148)	100 (50, 160)

Abbreviations: NC, not calculable; NR, not reached.

a Participants who had not received prior systemic therapy.

b Participants with disease progression on (durvalumab monotherapy, *n* = 52; T75 + D, *n* = 47; STRIDE, *n* = 43; and tremelimumab monotherapy, *n* = 30), were intolerant to (durvalumab, *n* = 15; T75 + D, *n* = 10; STRIDE, *n* = 12; and tremelimumab monotherapy, *n* = 14) or refused (durvalumab monotherapy, *n* = 37; T75 + D, *n* = 27; STRIDE, *n* = 20; and tremelimumab monotherapy, *n* = 25) sorafenib.

c The median for duration of follow-up is the arithmetic median. The duration of follow-up was calculated from the date of randomization (part 2A and part 3) or the date of first study treatment dose (part 2B and part 4).

d DoR is the time from the first documentation of a confirmed complete response/partial response until the date of disease progression, death, or the last evaluable RECIST assessment.

e Calculated using the Kaplan–Meier technique. Analysis data cutoff: November 06, 2020.

### Response

In the clinically evaluable population, the highest confirmed ORR by BICR according to RECIST v1.1 across treatment arms occurred in the STRIDE (24.0%) and durvalumab plus bevacizumab (21.3%) arms (Table 1). The ORRs in the immunoSEQ-evaluable population were similar to those of the clinically evaluable population across arms (Table 1), albeit lower in the biomarker-evaluable populations for T75 + D and tremelimumab. The median duration of follow-up in censored participants was longer for parts 2 and 3 (durvalumab monotherapy, tremelimumab monotherapy, T75 + D, and STRIDE arms) than for part 4 (durvalumab plus bevacizumab arm; Table 1). The median DoR from onset of response by BICR according to RECIST v1.1 was 14.95 months for durvalumab monotherapy, 18.43 months for STRIDE, 23.95 months for tremelimumab monotherapy, and 13.21 months for T75 + D (Table 1). In part 4 (durvalumab plus bevacizumab), for participants who had an objective response, the confirmed median DoR from the onset of response based on BICR according to RECIST v1.1 (in the full-analysis set) was not reached.

### T-cell clonality

**Figure 1.**
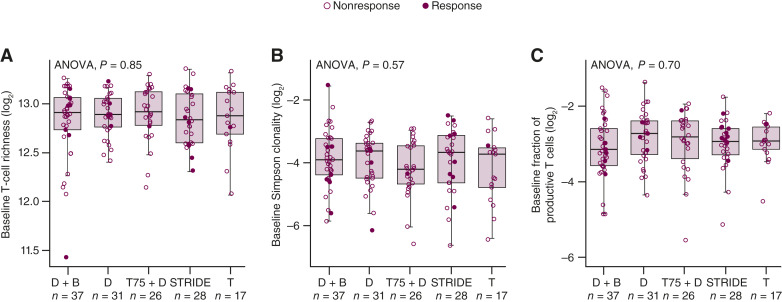
Baseline richness (**A**), Simpson clonality (**B**), and fraction of productive T cells (**C**) across the durvalumab plus bevacizumab, durvalumab monotherapy, T75 + D, STRIDE, and tremelimumab monotherapy arms. Each panel displays a distinct T-cell characteristic measured at baseline: (**A**) T-cell richness, (**B**) Simpson clonality, and (**C**) fraction of productive T cells. The *y*-axes represent each metric, whereas the *x*-axes indicate the treatment arms, with sample size for each arm included. One-way ANOVA performed for each panel showed no statistically significant differences among treatment arms at baseline. The lower and upper hinges of each boxplot are at the first and third quartiles. Boxplot whiskers extend to the most extreme value no further than 1.5 × IQR. Horizontal bar in each boxplot is at the median value. Analysis data cutoff: November 06, 2020. D, durvalumab monotherapy; T, tremelimumab monotherapy.

First, we evaluated baseline features to determine whether there were underlying differences in T cells that differentiated the treatment arms. These included (i) baseline richness, which reflects the variety of TCRs present, (ii) Simpson clonality, which measures how evenly the T-cell clones are distributed on a scale of 0 to 1, with 0 representing an even distribution of frequencies and 1 representing few clones at high frequencies, and (iii) the fraction of productive T cells (T cells that have undergone an in-frame TCR-β chain rearrangement such that they can be transcribed and translated into a functional protein). There were no significant differences in these features at baseline across treatment arms (Fig. 1).

**Figure 2.**
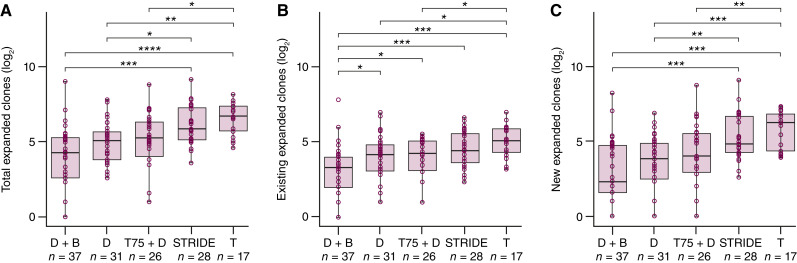
Median number of (**A**) total, (**B**) existing, and (**C**) new expanded T-cell clones at the end of cycle 1 across the durvalumab plus bevacizumab, durvalumab monotherapy, T75 + D, STRIDE, and tremelimumab monotherapy arms. A pairwise Wilcoxon rank-sum test performed on all available data per arm; *P* values adjusted for multiple comparisons using the Benjamini–Hochberg procedure. Significant differences between treatment arms are notated as follows: *, 0.05 ≥ *P* > 0.01; **, 0.01 ≥ *P* > 0.001; ***, 0.001 ≥ *P* > 0.0001; ****, 0.0001 ≥ *P* . Nonsignificant differences between treatment arms are unlabeled. The lower and upper hinges of each boxplot are at the first and third quartiles. Boxplot whiskers extend to the most extreme value no further than 1.5 × IQR. Horizontal bar in each boxplot is at the median value. Analysis data cutoff: November 06, 2020. D, durvalumab monotherapy; T, tremelimumab monotherapy.

Next, we evaluated whether T-cell features changed with treatment at the end of cycle 1 and observed a tremelimumab dose-dependent increase in the median number, as well as the 25^th^ and 75^th^ percentiles, of expanded T-cell clones in tremelimumab-containing arms versus durvalumab monotherapy (Table 1). An increase in the median number of total expanded T-cell clones versus durvalumab monotherapy was observed with STRIDE but was unchanged with durvalumab plus bevacizumab (Fig. 2A; Table 1). In addition, the greater expansion of T-cell clones with STRIDE versus durvalumab monotherapy seemed to be driven by new clones (Fig. 2C) rather than existing clones (Fig. 2B). The durvalumab monotherapy and durvalumab plus bevacizumab arms demonstrated the lowest median and 25^th^ and 75^th^ percentile number of expanded T-cell clones after cycle 1 (Table 1), with durvalumab plus bevacizumab showing a reduction in existing expanded clones relative to durvalumab (Fig. 2B). Other statistically significant changes at the end of cycle 1 versus baseline included an increase in richness with tremelimumab (*P* = 0.038) and a decrease in Simpson clonality with STRIDE (*P* = 0.015) and tremelimumab (*P* = 0.017; Supplementary Fig. S1A and S1B). In addition, a statistically significant decrease in the fraction of productive T cells was observed with durvalumab plus bevacizumab (*P* = 0.015), and a statistically significant increase was observed with tremelimumab (*P* = 0.004; Supplementary Fig. S1C). The decrease in the fraction of productive T cells with durvalumab plus bevacizumab was the only change among these three features that showed significant differences between responders and nonresponders (Supplementary Table S4), with responders showing an increase whereas nonresponders showed a decrease (Supplementary Fig. S1D).

**Figure 3.**
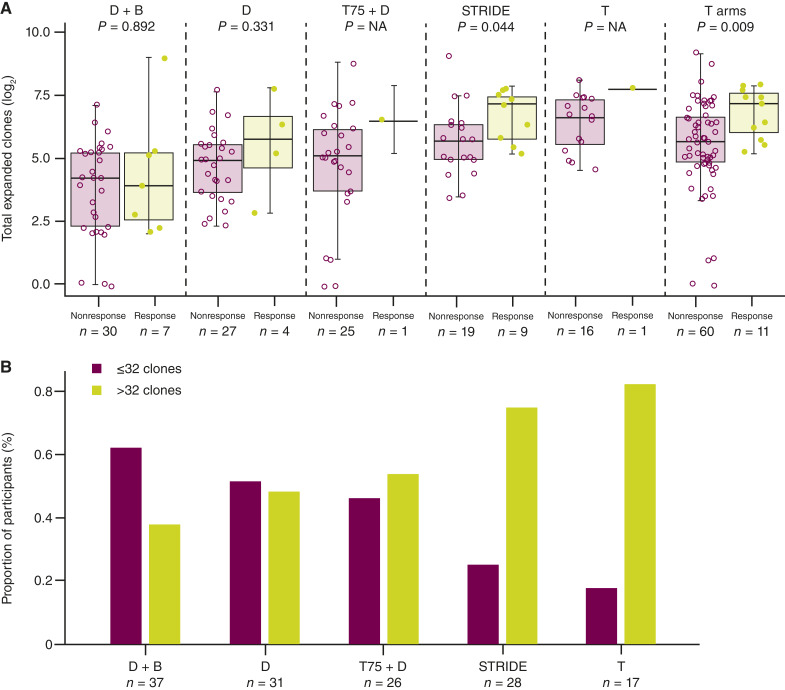
**A**, Median number of total expanded T-cell clones by response across the durvalumab plus bevacizumab, durvalumab monotherapy, T75 + D, STRIDE, and tremelimumab monotherapy arms, and all of the tremelimumab-containing arms combined. **B**, Proportion of participants with >32 expanded T-cell clones in the durvalumab plus bevacizumab, durvalumab monotherapy, T75 + D, STRIDE, and tremelimumab monotherapy arms. For panel **A**, *P* values were derived from a Wilcoxon test comparing response arms. Statistical analysis included nonevaluable participants (zero value total expanded T-cell clones). The lower and upper hinges of each boxplot are at the first and third quartiles. Boxplot whiskers extend to the most extreme value no further than 1.5 × IQR. Horizontal bar in each boxplot is at the median value. For panel **B**, the proportion of responders who either had more or less than or equal to the durvalumab median of 32 clones is shown. Analysis data cutoff: November 06, 2020. D, durvalumab monotherapy; NA, not available; T, tremelimumab monotherapy.

When we explored a possible association between the increase in expanded T-cell clones and clinical response, responders had a larger median number of expanded clones than nonresponders in tremelimumab-containing arms; there was no relationship between expanded clones and response in the durvalumab monotherapy or durvalumab plus bevacizumab arms (Fig. 3A). This trend held true when considering individual tremelimumab-containing arms, although the T75 + D and tremelimumab monotherapy arms had limited numbers of responders with evaluable samples. In addition, the proportion of participants in tremelimumab-containing arms whose median number of expanded clones exceeded the median for durvalumab monotherapy (i.e., >32 expanded clones) increased with increasing tremelimumab dose (Fig. 3B).

These results suggest that tremelimumab, but not bevacizumab, when combined with durvalumab leads to an increase in the number of expanded clones versus durvalumab monotherapy. In addition, although sample size precludes robust conclusions, it seems that for each tremelimumab-containing arm, response rates in the biomarker-evaluable population were higher for participants whose median number of expanded clones exceeded the median of the durvalumab arm (Supplementary Table S5).

### Changes in gene expression

**Figure 4.**
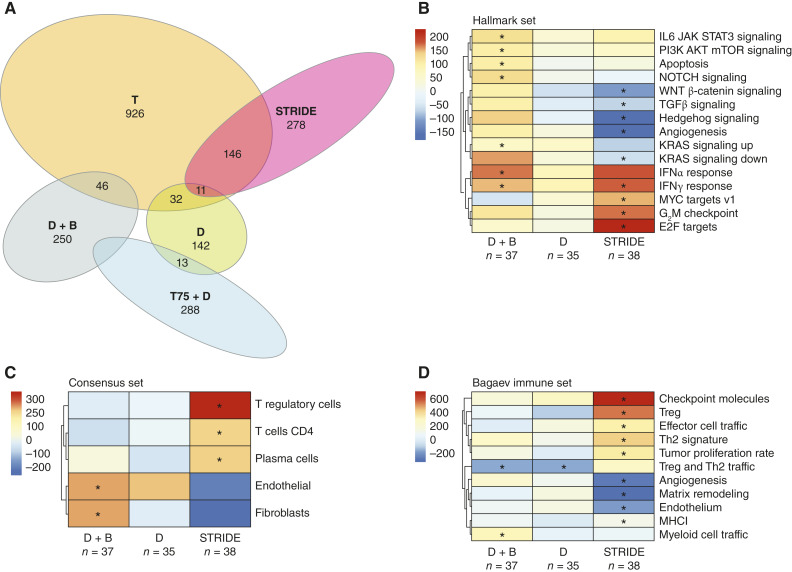
Gene expression. Peripheral blood gene expression changes across the durvalumab plus bevacizumab, durvalumab monotherapy, T75 + D, STRIDE, and tremelimumab monotherapy arms (**A**) and changes in gene expression signatures in the Hallmark set (**B**), the consensus tumor microenvironment set (**C**), and the Bagaev immune set (**D**) across the durvalumab plus bevacizumab, durvalumab monotherapy, and STRIDE arms. In panel **A**, to identify differential gene expression in each trial arm, the DESeq2 capability was applied in R to compare the end of cycle 1 with day 1, while adjusting for baseline participant effects. A Venn diagram comparing differential gene expression per trial arm was produced according to the following criteria: |log_2_ fold change| ≥ 0.4 and *P* ≤ 0.05 (*P* values are unadjusted; no multiple hypothesis testing applied at this stage). In panels **B–D**, a mixed effects model accounting for random patient effects was applied to the cycle 1 vs. baseline change in signature expression, calculated independently per arm. The values shown in the legends of panels **B–D** are the estimated slope of the linear regression, comparing the end of cycle 1 with the baseline for each gene expression signature tested. The resulting *P* values of the test were used to select the signatures illustrated in the heatmap. *, *P* ≤ 0.05. D, durvalumab monotherapy; T, tremelimumab monotherapy; Treg, regulatory T cell.

Distinct, and largely nonoverlapping, peripheral blood gene expression changes were observed for the different treatment arms (Fig. 4A). Overall, more gene expression changes in peripheral blood were observed for tremelimumab-containing and combination regimens than for durvalumab monotherapy, with tremelimumab monotherapy and STRIDE showing the greatest number of changes (Fig. 4A; Supplementary Table S6).

**Figure 5.**
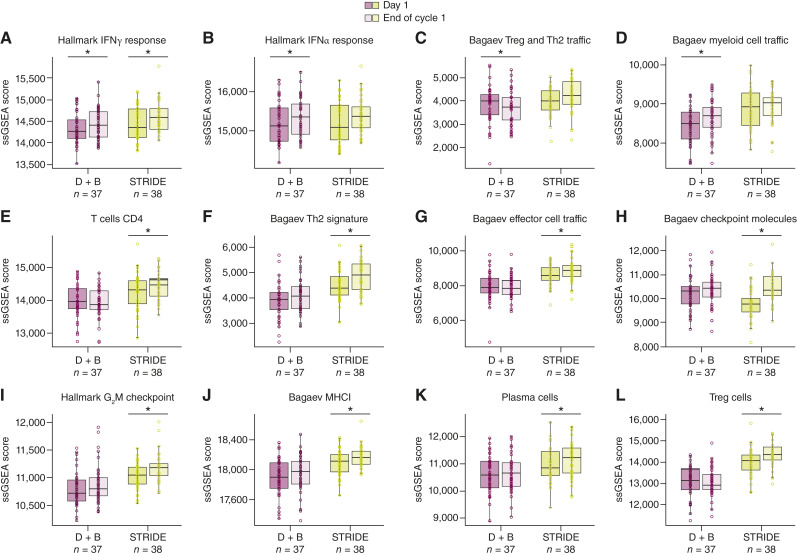
Gene expression signatures. **A–L,** Selected gene expression signature changes for STRIDE and durvalumab plus bevacizumab. To evaluate specific changes in gene expression signatures across treatment arms, selected gene expression signatures are shown as boxplots. *P* values were derived from a mixed-effect linear model adjusted for random participant effects, comparing the end of cycle 1 with day 1 separately for each trial arm, D + B, and STRIDE. *, *P* ≤ 0.05. Panel **A**, *P* ≤ 0.05 for both D + B and STRIDE; panels **B–D,***P* ≤ 0.05 for D + B; and panels **E–L,***P* ≤ 0.05 for STRIDE. The lower and upper hinges of each boxplot are at the first and third quartiles. Boxplot whiskers extend to the most extreme value no further than 1.5 × IQR. Horizontal bar in each boxplot is at the median value. Analysis data cutoff: November 06, 2020. MHCI, MHC class I; ssGSEA, single-sample gene set enrichment analysis; Treg, regulatory T cell.

In a side-by-side comparison focusing on immunomodulatory gene expression signatures for combination treatments, both STRIDE and durvalumab plus bevacizumab elicited more changes in immunomodulatory gene expression signatures than durvalumab alone (Fig. 4B–D). However, more and a greater magnitude of changes in gene expression signatures were observed with STRIDE than with durvalumab plus bevacizumab (Fig. 4B–D; Supplementary Table S6). Interestingly, most changes in gene expression signatures did not overlap between STRIDE and durvalumab plus bevacizumab (Figs. 4B–D and 5; Supplementary Fig. S2; Supplementary Table S6). Although both STRIDE and durvalumab plus bevacizumab elicited IFNγ signature upregulation, IFNα response, myeloid cell and endothelial signatures were only upregulated by durvalumab plus bevacizumab (Figs. 4C and 5). In contrast, STRIDE upregulated several gene expression signatures associated with proliferating T-effector cells, including G2M and *E2F*, and CD4^+^ T cells and effector cell trafficking signatures (Figs. 4B and 5; Supplementary Fig. S2). In addition, upregulation in T-regulatory cell, Th2, and checkpoint molecule gene expression signatures observed with STRIDE seemed to be dependent on the tremelimumab dose (Supplementary Fig. S2), consistent with previously reported mechanisms of CTLA-4 blockade (22, 23). STRIDE also upregulated individual genes associated with CTLA-4 and PD-(L)1 blockade that are components of several signatures, including CTLA-4, PDCD1, RGS1, CD28, ICOS, and MHCI components (Supplementary Tables S7 and S8; refs. 17–19).

## Discussion

Results from Study 22 and phase III studies support the efficacy of the dual ICI STRIDE regimen and anti–PD-L1 antibodies plus bevacizumab for treating uHCC (1, 2, 8). This current analysis explored the potential for distinct immunologic mechanisms by which these regimens elicit improved clinical activity beyond ICI monotherapy.

### TCR clonality

Differing results in the number and nature of expanded T-cell clones for durvalumab plus bevacizumab versus tremelimumab-containing regimens suggest different mechanisms of action for these treatments. It has been hypothesized that the single 300 mg dose of tremelimumab in the STRIDE regimen primes an immune response via activation and proliferation of both CD4^+^ and CD8^+^ T-cell populations that are then sustained by subsequent durvalumab monotherapy to avoid immune tolerance (24). In support of this model, we previously showed that the STRIDE regimen produces the greatest expansion of CD8^+^ Ki67^+^ T cells, which was associated with clinical response (8). The data from this study build upon those initial findings (10, 24) and show a substantial expansion of T-cell clones with STRIDE that is associated with clinical response, with the larger T-cell pool resulting from dual immune checkpoint inhibition comprising both existing and newly primed subsets. The findings from this study and the model for T-cell expansion with STRIDE are supported by similar work in melanoma. In one such study with paired samples of single-cell RNA and TCR sequencing analysis of participants receiving ICI therapy, anti–CTLA-4 was shown to induce strong expansion and proliferation of progenitor-exhausted CD8^+^ T-cell clones, which were then reinvigorated by anti–PD-1 during combination therapy (25). The statistically significant decrease in Simpson clonality (STRIDE and tremelimumab arms) and increase in richness in the tremelimumab arm are consistent with a tremelimumab-dependent increase in expanded T-cell clones, suggesting that higher doses of tremelimumab contribute to a large variety of T-cell clones with a more even distribution of frequencies.

Collectively, our findings are consistent with previously reported studies and highlight the potential of T-cell clonal expansion in the periphery to serve as a biomarker for predicting long-term responses to ICIs (26, 27). These findings also provide a plausible mechanism underlying the durability of response and association between response and long-term survival in patients treated with CTLA-4–containing ICI combinations in HCC (28–30).

In contrast, no association between the expansion of T-cell clones and clinical response was observed for durvalumab plus bevacizumab. Expansion of T-cell clones was similar between durvalumab plus bevacizumab and durvalumab monotherapy, and there was no association of T-cell clonal expansion with objective response, suggesting that the efficacy of the combination with bevacizumab may be due to boosting an already inflamed phenotype in the tumor, rather than the expansion of new T-cell clones that could be detectable in the periphery. These results are indicative of similar observations from an integrated molecular analysis of participants with advanced HCC from the phase Ib GO30140 and phase III IMbrave150 studies, which showed that tumor samples with preexisting immunity (high expression of CD274 mRNA, T-effector gene expression signature, and intratumoral CD8^+^  T -cell density) and high PD-L1 protein expression were associated with clinical benefit from treatment with atezolizumab plus bevacizumab (13).

Taken together, these data suggest that STRIDE, but not durvalumab plus bevacizumab, may elicit durable clinical responses by expanding the number of unique T-cell clones and increasing T-cell proliferation beyond those observed with durvalumab monotherapy.

### RNA-seq results

Both STRIDE and durvalumab plus bevacizumab elicited changes in immune gene expression and shared an increase in IFNγ response gene expression signatures, but otherwise demonstrated mostly different immunomodulatory changes detectable in peripheral blood. These changes, which generally exceeded those of durvalumab monotherapy, suggest that each combination has a greater capacity for immunomodulation than durvalumab monotherapy and support the concept that adding bevacizumab or tremelimumab to durvalumab elicits a more profound immune effect that results in increased clinical response rates.

Both the number and nature of gene expression changes suggest that the dual ICI STRIDE regimen induced a robust immune response in participants with uHCC that was necessary and sufficient to drive an immune and clinical response greater than durvalumab monotherapy alone, as reported previously (10, 24). STRIDE’s upregulation of proliferation (G2M/*E2F*), CD4 T-cell activation, and effector cell trafficking signatures in the blood is consistent with flow cytometry results reported for Study 22 and previously reported mechanisms of CTLA-4 blockade (8, 22, 31, 32). Combined with the increase in expanded T-cell clones, this supports the mechanistic hypothesis that the STRIDE regimen activates and recruits T cells from the tumor-draining lymph nodes to the tumor (8, 22).

Although the most robust changes in peripheral blood T-cell clone expansion and gene expression were observed with tremelimumab-containing therapies, interesting and distinct trends in immune gene expression signatures were observed with durvalumab plus bevacizumab. Consistent with the hypothesized mechanism of action for bevacizumab (33), IFNα response and myeloid cell signatures were upregulated by durvalumab plus bevacizumab. The upregulation of the endothelial and Notch signaling gene expression signatures observed with durvalumab plus bevacizumab is consistent with findings that suggest potential compensatory pathway upregulation with VEGF inhibition (34). The increase in the fibroblast signature has not been fully characterized. Although cancer-associated fibroblasts and tumor endothelial cells have previously been found in circulation, this finding requires further investigation (35, 36).

These findings, as well as the relatively lower degree of T-cell expansion detectable in the periphery, are consistent with a proposed local mechanism of action of VEGF blockade that includes normalization of blood vessels in the tumor, maturation of dendritic cells, and repolarization of tumor-associated macrophages to an immunostimulatory phenotype that results in enhanced CD8^+^ T-cell infiltration into tumors (12, 37, 38). Indeed, in an earlier disease setting in which locoregional therapies, such as transarterial chemoembolization (TACE), are part of standard global treatment paradigms, recent outcomes in the phase III EMERALD-1 and LEAP-012 studies suggest that PD-(L)1 plus VEGF blockade combination therapy may improve progression-free survival compared with TACE alone (1, 2, 5, 39, 40).

This analysis has several limitations, owing to its exploratory nature. The percentage of patients with paired samples for analysis was similar across arms, suggesting the absence of systematic bias. Another limitation is that the durvalumab monotherapy and tremelimumab-containing arms enrolled a predominantly second-line population with disease progression on, intolerance to, or refusal of prior sorafenib. Conversely, patients allocated to the durvalumab plus bevacizumab arm were treatment naïve. However, supporting the comparability of immune responses between arms, ORRs with ICI regimens, including STRIDE in both the first- and second-line settings, have been largely consistent and line-agnostic in ICI-naïve HCC populations (41). Furthermore, the lack of significant differences in baseline TCR measures and gene expression signature profiles in all arms suggests that the observed changes in immune profiles in this study are likely representative of ICI-naïve populations, whether in the first- or second-line setting (1, 2, 8, 42–44). Another potential limitation was the difference in cycle length between arms, with the end of cycle 1 being day 29 for the durvalumab and tremelimumab arms and day 22 for the durvalumab plus bevacizumab arm. The optimal timepoint for assessment of immune cell changes in response to the therapy is not known and may vary according to regimen. Although a potential delayed expansion contributing to a clinical response cannot be discounted, time to response with ICI combinations is generally short. A median time to response of approximately 2 months has been reported for the STRIDE, durvalumab, atezolizumab plus bevacizumab, and nivolumab plus ipilimumab regimens (1, 28, 45). Long-term follow-up of peripheral blood samples was not obtained in this study, so it remains unknown whether later expansion of clones or changes in immune response contributed to the measured response. A small subset of patients may also experience delayed responses or initial progression, followed by subsequent response, which would not be captured because of the constraints of the samples available for this biomarker analysis. Further supporting the comparability between arms, previous work with durvalumab plus tremelimumab has shown that changes in peripheral blood cell populations persist qualitatively at days 21 and 29 following initial treatment (46). In addition, other analyses of transcriptomic and T-cell clonality changes have focused on day 21 (27). A final limitation was the lack of on-treatment tumor tissue to determine whether peripheral immune cell changes on treatment are reflective of corresponding alterations in the tumor microenvironment.

In conclusion, STRIDE and durvalumab plus bevacizumab seem to function through largely independent and distinct mechanisms of action to achieve clinical responses greater than those of durvalumab monotherapy.

These findings support the hypothesis that if STRIDE and bevacizumab or other VEGF-targeted therapies could be combined safely, they may function via complementary pathways to improve clinical response in uHCC. Furthermore, these data may be useful when considering optimal dosing and sequencing of immunotherapy and antiangiogenic therapies.

## Supplementary Material

Supplementary Data Supplementary Material

Supplementary Table S6 Supplementary Table S6

## Data Availability

Data underlying the findings described in this article may be obtained in accordance with AstraZeneca’s data-sharing policy described at https://astrazenecagrouptrials.pharmacm.com/ST/Submission/Disclosure. Data for studies directly listed on Vivli can be requested through Vivli at www.vivli.org. Data for studies not listed on Vivli can be requested through Vivli at https://vivli.org/members/enquiries-about-studies-not-listed-on-the-vivli-platform/. The AstraZeneca Vivli member page is also available, outlining further details at https://vivli.org/ourmember/astrazeneca/.
