# Supplementary material for: T-Cell Receptor and Immune Gene Expression Pharmacodynamics for Durvalumab Alone and with Tremelimumab or Bevacizumab in Unresectable Hepatocellular Carcinoma
Source: Clin Cancer Res. 2025 Dec 8;32(4):694–704. doi: 10.1158/1078-0432.CCR-25-1592 (PMC13056246; doi:10.1158/1078-0432.CCR-25-1592)
Supplement: Supplementary Data — Supplementary Material [file ccr-25-1592_supplementary_data_suppsm.pdf]

SUPPLEMENTAL MATERIAL

Plain Language Summary

Why did we perform this research?

Hepatocellular carcinoma (HCC) is the most common type of liver cancer. Tremelimumab and durvalumab are types of medications called immunotherapies that help the body's immune system to fight cancer. T cells are an important group of white blood cells that play a vital role in fighting cancer. STRIDE is a treatment that combines a single dose of tremelimumab with monthly doses of durvalumab. Bevacizumab is a medication that reduces blood flow to the tumor, promotes movement of T cells into tumors, and increases immune response in tumors. Study 22 is a phase I/II clinical study that investigated durvalumab alone or in combination with tremelimumab or bevacizumab in liver cancer. Previous results from Study 22 showed that a higher percentage of participants who took STRIDE had decreased size or number of tumors than those who took durvalumab alone. Similar results were observed in participants who took durvalumab plus bevacizumab compared with those who took STRIDE. We performed this research to look at how these treatments affected the immune response from T cells and gene expression (the process where information from genes is used to make functional products such as proteins), which could be related to how well the treatments worked.

How did we perform this research?

We looked at the increase in amounts of certain types of T cells and the changes in gene expression in blood samples between the start of therapy and the end of the first treatment cycle.

What were the findings of this research?

STRIDE, but not durvalumab plus bevacizumab, was associated with an expansion of the number of specific T cell clones compared with durvalumab alone, and this effect was associated with a decrease in the extent of tumors after treatment. Both STRIDE and durvalumab plus bevacizumab showed evidence of an increased immune response based on gene expression changes in blood samples compared with durvalumab alone, though the changes in immune-related genes generally differed for STRIDE and durvalumab plus bevacizumab.

What are the implications of this research?

These results suggest that STRIDE and durvalumab plus bevacizumab have different ways of working and may be able to work together to fight cancer in HCC.

Supplementary Figures

Figure S1. Change from baseline at the end of Cycle 1 in (A) richness, (B) Simpson clonality, and (C) fraction of productive T cells across the durvalumab plus bevacizumab, durvalumab monotherapy, T75+D, STRIDE, and tremelimumab monotherapy arms. (D) Change from baseline at the end of Cycle 1 in fraction of productive T cells with response and non-response shown separately across treatment arms.

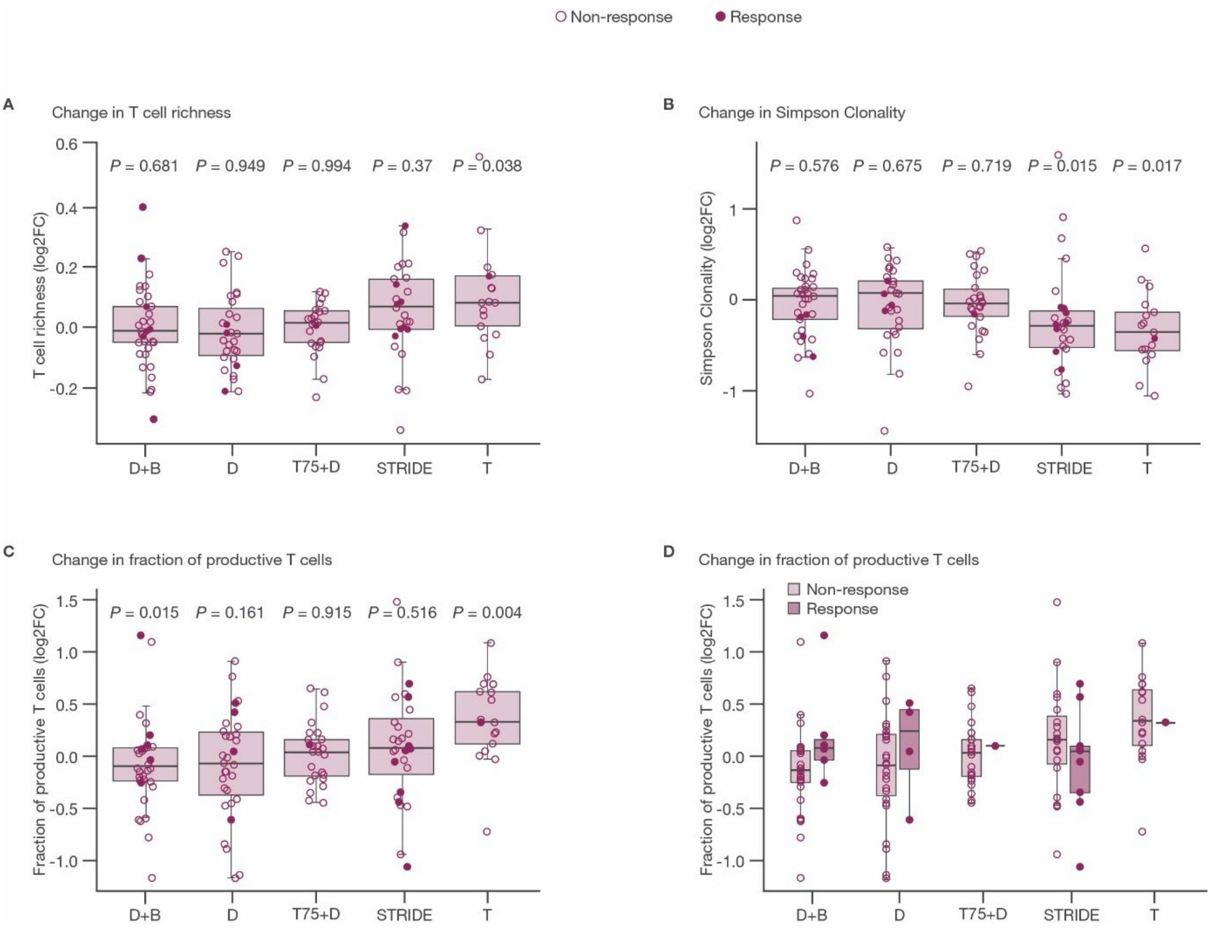

Each panel displays the log2 fold change from baseline in a distinct T cell parameter: (A) T cell richness, (B) Simpson clonality, and (C) and (D) fraction of productive T cells. The y-axes indicate the $\log_2$ fold change, and the x-axes denote the treatment arms, with sample size shown for each arm. *P* values for each treatment arm are derived from a mixed-effects linear regression model comparing end of Cycle 1 to baseline values. Participants are color-coded according to response status (responders and non-responders). Response status is plotted separately in panel D to emphasize the response-associated treatment effect with D+B noted in Table S3.

Figure S2. Heatmaps showing changes in gene expression signatures in (**A**) the Hallmark set, (**B**) the consensus TME set, and (**C**) the Bagaev immune set across the durvalumab plus bevacizumab, durvalumab monotherapy, T75+D, STRIDE, and tremelimumab monotherapy arms.

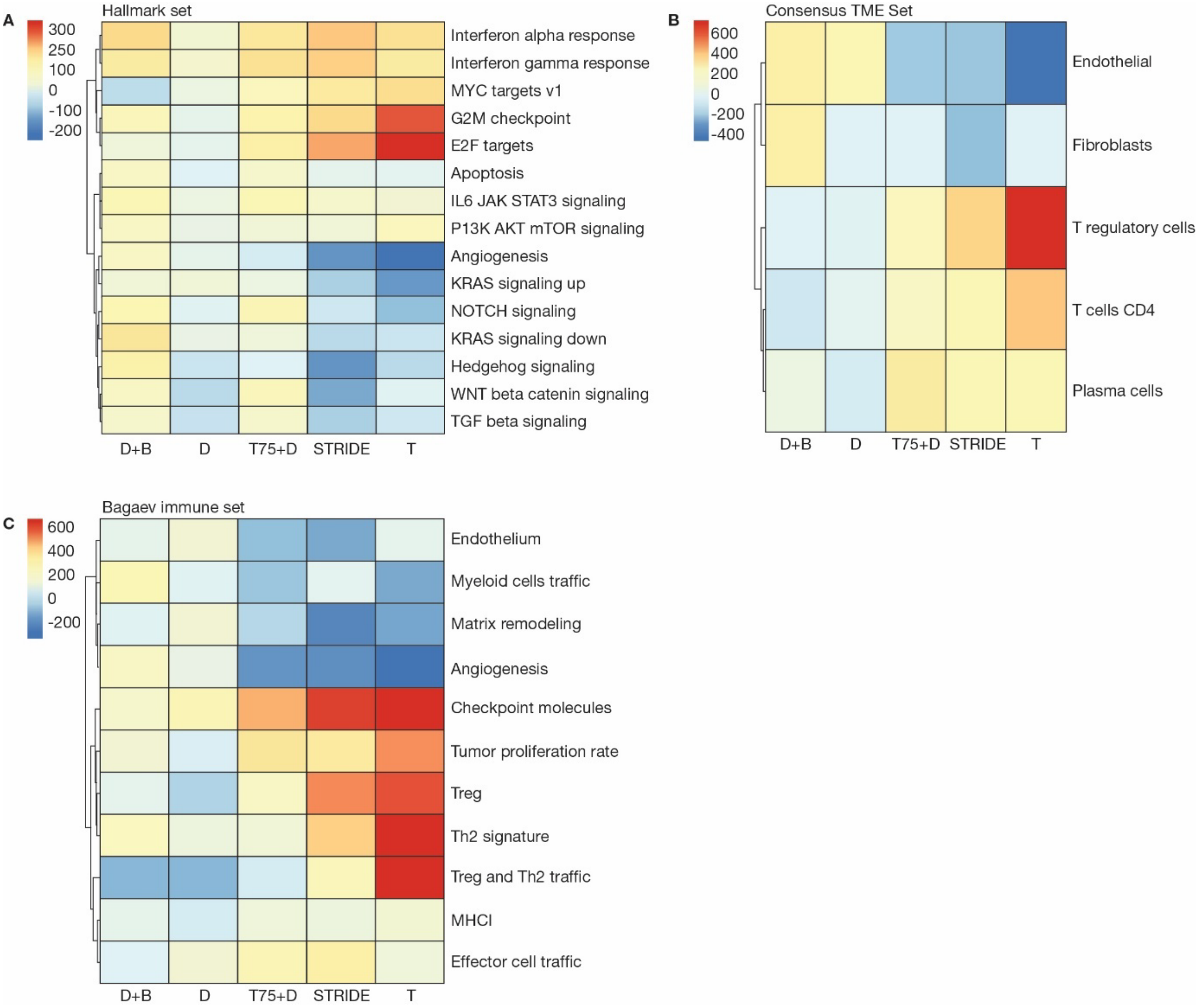

A mixed effects model accounting for random patient effects was applied to the Cycle 1 vs Baseline change in signature expression, calculated independently per arm. The values shown in the legends of panels A, B, and C are the estimated slope of the linear regression, comparing the end of Cycle 1 to the baseline for each gene expression signature tested. AKT, Ak strain transforming; CD4, cluster of differentiation 4; D, durvalumab monotherapy; D+B, durvalumab plus bevacizumab; IL6, interleukin 6; KRAS, Kirsten rat sarcoma virus; log2FC, log2 fold change; MYC, myelocytomatosis viral oncogene homolog; MHC1, major histocompatibility complex class I; MTOR, mammalian target of rapamycin; PI3K, phosphoinositide 3-kinases; STRIDE, Single

Tremelimumab Regular Interval Durvalumab; T, tremelimumab monotherapy; T75+D, tremelimumab 75 mg plus durvalumab; TGF, transforming growth factor; Th2, T helper 2; TME, tumor microenvironment; Treg, regulatory T cells; UV, ultraviolet; WNT, wingless type.

Supplementary Tables

Table S1. Participant demographics and baseline characteristics across the durvalumab monotherapy, T75+D, STRIDE, and tremelimumab monotherapy arms**.**

**Characteristic**	**Durvalumab monotherapy (*n* = 104)**	**T75+D (*n* = 84)**	**STRIDE (*n* = 75)**	**Tremelimumab monotherapy (*n* = 69)**
Median age, years (range)	64.5 (32–89)	61.5 (28–82)	66.0 (26–86)	62.0 (37–81)
Sex, male, *n* (%)	92 (88.5)	70 (83.3)	65 (86.7)	57 (82.6)
Race and ethnicity, *n* (%)				
White	35 (33.7)	30 (35.7)	27 (36.0)	26 (37.7)
Black	10 (9.6)	5 (6.0)	4 (5.3)	2 (2.9)
Asian	55 (52.9)	47 (56.0)	44 (58.7)	39 (56.5)
Hispanic or Latino	5 (4.8)	5 (6.0)	4 (5.3)	4 (5.8)
Others	4 (3.8)	2 (2.4)	0	2 (2.9)
ECOG PS, n (%)				
0	52 (50.0)	51 (60.7)	46 (61.3)	45 (65.2)
1	52 (50.0)	33 (39.3)	29 (38.7)	24 (34.8)
Child-Pugh score, *n* (%)				
A/5	79 (76.0)	54 (64.3)	51 (68.0)	44 (63.8)
A/6	23 (22.1)	26 (31.0)	23 (30.7)	24 (34.8)
B/7	2 (1.9)	4 (4.8)	1 (1.3)	1 (1.4)
BCLC score, *n* (%)				
A	1 (1.0)	1 (1.2)	1 (1.3)	2 (2.9)
B	9 (8.7)	17 (20.2)	13 (17.3)	13 (18.8)
C	80 (76.9)	57 (67.9)	58 (77.3)	42 (60.9)

Unknown or missing[a]	14 (13.5)	9 (10.7)	3 (4.0)	12 (17.4)
Extent of disease, *n* (%)				
Macrovascular invasion	30 (28.8)	20 (23.8)	16 (21.3)	17 (24.6)
Extrahepatic disease	63 (60.6)	48 (57.1)	53 (70.7)	31 (44.9)
AFP, ≥400 ng/mL, *n* (%)	39 (37.5)	34 (40.5)	35 (46.7)	33 (47.8)
Viral status, *n* (%)				
HBV infection	40 (38.5)	29 (34.5)	27 (36.0)	27 (39.1)
HCV infection	28 (26.9)	26 (31.0)	21 (28.0)	20 (29.0)
Uninfected	36 (34.6)	29 (34.5)	27 (36.0)	22 (31.9)
PD-L1 status, *n* (%)				
TC/IC ≥1%[b]	55 (52.9)	41 (48.8)	27 (36.0)	40 (58.0)
TC/IC <1%	35 (33.7)	31 (36.9)	38 (50.7)	24 (34.8)
Missing	14 (13.5)	12 (14.3)	10 (13.3)	5 (7.2)
Prior sorafenib therapy, *n* (%)				
Progressed	52 (50.0)	47 (56.0)	43 (57.3)	30 (43.5)
Intolerant[c]	15 (14.4)[c]	10 (11.9)[c]	12 (16.0)	14 (20.3)
Refused	37 (35.6)	27 (32.1)	20 (26.7)	25 (36.2)
Previous treatment modalities, *n* (%)				
Systemic therapy	66 (63.5)	55 (65.5)	55 (73.3)	44 (63.8)
Radiation	16 (15.4)	22 (26.2)	22 (29.3)	15 (21.7)
Cancer-related surgery	37 (35.6)	37 (44.0)	34 (45.3)	23 (33.3)

Table originally published in Kelley RK, et al. Safety, efficacy, and pharmacodynamics of tremelimumab plus durvalumab for patients with unresectable hepatocellular carcinoma: randomized expansion of a phase I/II study. *J Clin Oncol* 2021;39:2995. https://ascopubs.org/doi/full/10.1200/JCO.20.03555. Reproduced with permission.

AFP, alpha-fetoprotein; BCLC, Barcelona Clinic Liver Cancer; ECOG PS, Eastern Cooperative Oncology Group performance status; HBV, hepatitis B virus; HCV, hepatitis C virus; IC, immune cell; PD-L1, programmed cell death ligand-1; STRIDE, Single Tremelimumab Regular Interval Durvalumab; T75+D, tremelimumab 75 mg plus durvalumab; TC, tumor cell; TNM, tumor, node, metastasis.

[a]At the start of the study, the Protocol required the use of modified TNM staging together with the fibrosis score. The study was amended to include BCLC at a later date. Thus, BCLC scores are missing for some participants.

[b]Defined as PD-L1 staining of any intensity in TC membranes and/or tumor-associated ICs in the tumor area.

[c]Includes three participants (durvalumab monotherapy, $n = 1$; T75+D, $n = 2$) confirmed with a documented contraindication to sorafenib. These participants were not offered sorafenib and could not refuse treatment. These participants are captured as intolerant, although sorafenib therapy was not recorded.

Table S2. Participant demographics and baseline characteristics in the durvalumab plus bevacizumab arm**.**

**Characteristic**	**Durvalumab plus bevacizumab (*n* = 47)**
Median age, years (range)	64.0 (37–84)
Sex, male, *n* (%)	41 (87.2)
Race and ethnicity, *n* (%)	
White	7 (14.9)
Black	1 (2.1)
Asian	39 (83.0)
Hispanic or Latino	0
Others	0
ECOG PS, n (%)	
0	21 (44.7)
1	26 (55.3)
Child-Pugh score, *n* (%)	
A/5	41 (87.2)
A/6	6 (12.8)
B/7	0
BCLC score, *n* (%)	
A	8 (17.0)
B	9 (19.1)
C	21 (44.7)
Unknown or missing[a]	7 (14.9)
Extent of disease, *n* (%)	
Macrovascular invasion	19 (40.4)
Extrahepatic disease	30 (63.8)
AFP, ≥400 ng/mL,	17 (36.2)

n (%)	
Viral status, *n* (%)	
HBV infection	24 (51.1)
HCV infection	6 (12.8)
Uninfected	17 (36.2)
PD-L1 status, *n* (%)	
TC/IC ≥1%[b]	22 (46.8)
TC/IC <1%	20 (42.6)
Missing	5 (10.6)
Prior sorafenib therapy, *n* (%)	
Progressed	0
Intolerant	0
Refused	0
Previous treatment modalities, *n* (%)	
Systemic therapy	0
Radiation	16 (34.0)
Cancer-related surgery	9 (19.1)

AFP, alpha-fetoprotein; BCLC, Barcelona Clinic Liver Cancer; ECOG PS, Eastern Cooperative Oncology Group performance status; HBV, hepatitis B virus; HCV, hepatitis C virus; IC, immune cell; PD-L1, programmed cell death ligand-1; TC, tumor cell; TNM, tumor, node, metastasis.

[a]At the start of the study, the Protocol required the use of modified TNM staging together with the fibrosis score. The study was amended to include BCLC at a later date. Thus, BCLC scores are missing for some patients.

[b]Defined as PD-L1 staining of any intensity in TC membranes and/or tumor-associated ICs in the tumor area.

Table S3. Representativeness of trial participants in Study 22.

**Category**	**Details**
Disease, problem, or condition under investigation	Hepatocellular carcinoma (HCC)
Special consideration related to:	
Sex and gender	Incidence of primary liver cancer is 2 to 3 times higher in males than females globally (1).
Age	Peak incidence of HCC occurs at approximately 75 years of age (1).
Race and ethnic group	In the United States, the highest incidence of HCC has been observed in Asian/Pacific Islanders, and the lowest incidence rates have been observed in non-Hispanic whites (2).
Geography	Eastern Asian countries, including South Korea and Japan, have the highest reported rates of HCC (1,3).
Other considerations	Key etiologies of HCC include hepatitis infections, which may contribute to geographic and temporal trends in incidence (4).
Overall representativeness of this trial	Participants enrolled in the Study 22 trial were mostly male across all the treatment arms (83%–89%), reflecting the trend seen in the general patient population (1). The median age of participants across treatment arms ranged from 62–66 years. The median age was younger than the peak age of incidence reported in the literature (1,2). Most participants across treatment arms were of Asian race (53 –83%), consistent with geographic and race/ethnicity trends in literature (1). The percentage of participants positive for hepatitis B virus ranged from 35%–51%. The percentage of participants positive for hepatitis C virus ranged from 13%–29%.

Table S4. The association of change in richness, fraction of productive T cells, and Simpsons clonality from baseline with response.

	Durvalumab plus bevacizumab	Durvalumab monotherapy	T75+D	STRIDE	Tremelimumab monotherapy
**Richness**	0.591	0.188	0.990	0.564	0.578
**Fraction of productive T cells**	0.032	0.286	0.586	0.734	0.897
**Simpsons clonality**	0.147	0.691	0.722	0.579	0.594

STRIDE, Single Tremelimumab Regular Interval Durvalumab; T75+D, tremelimumab 75 mg plus durvalumab. Values represent *P* values of the interaction effect (Day × Responder status) derived from mixed effects linear regression analyses of Richness, Fraction of Productive T cells, and Simpson clonality in each treatment arm.

The regression model incorporates Day (baseline or end of Cycle 1), responder status (responder or non-responder), and their interaction, with individual patient identifier (ecode) included as a random effect. Interaction *P* values indicate whether changes from baseline to end of Cycle 1 differ significantly between responders and non-responders within each treatment arm.

Table S5. Proportion of participants with expanded clones by clinical response status in the biomarker-evaluable population across the durvalumab monotherapy, T75+D, STRIDE, and tremelimumab monotherapy arms.

	**Durvalumab monotherapy (*n* = 104)**	**T75+D (*n* = 84)**	**STRIDE (*n* = 75)**	**Tremelimumab monotherapy (*n* = 69)**
**Total participants in BEP, *n***	31	26	28	17
**Participants with > the durvalumab median of 32 clones, *n* (%)**	15/31 (48.39)	14/26 (53.85)	21/28 (75.00)	14/17 (82.35)
**Percentage of responders in those with ≤32 clones, *n* (%)**	1/16 (6.25)	0/12 (0)	0/7 (0)	0/3 (0)
**Percentage of responders in those with >32 clones, *n* (%)**	3/15 (20.00)	1/14 (7.14)	9/21 (42.86)	1/14 (7.14)

BEP, biomarker-evaluable population; STRIDE, Single Tremelimumab Regular Interval Durvalumab; T75+D, tremelimumab 75 mg plus durvalumab.

Table S6. Immune-related peripheral blood gene expression changes in the durvalumab plus bevacizumab, durvalumab monotherapy, T75+D, STRIDE, and tremelimumab monotherapy arms.

[Please see associated Excel file.]

Table S7. List of the genes that comprise the gene expression signature for the consensus TME set.

**T Regulatory cells**		**Endothelial cells**	**Fibroblasts**	**Plasma cells**	**T cells CD4**			
CCR3	GPR171	CD93	ASPN	AMPD1	ACAP1	CD96	GZMM	OSM
CCR7	ICOS	CDH5	COL14A1	CAMP	AIM2	CTLA4	ICOS	PIK3IP1
CD2	IL10RA	EMCN	COL3A1	CD19	ARHGAP15	CTSW	IFNG	PRMT2
CD247	IL2RA	ERG	DCN	CD27	BATF	CXCR6	IKZF1	PTGER2
CD27	IL2RB	KDR	GREM1	CD38	BCL11B	DPEP2	IL10RA	PTPRCAP
CD37	ITGB7	PCDH12	ISLR	CD79A	CCL4	FAIM3	IL16	PVRIG
CD3E	ITK	RAMP3	LMOD1	CD79B	CCR2	FAM65B	IL2RA	RGS1
CD5	LCK	TFEC	PRKG1	CYBA	CCR7	FCN1	IL2RB	SAMSN1
CD7	LTB		SGCD	DOK3	CD2	GIMAP4	ITGB7	SELPLG
CTLA4	PIK3IP1		TCF21	ENTPD1	CD247	GIMAP6	ITK	SLAMF1
CXCR6	RGS1		WISP1	FCRL2	CD27	GPR132	JAK3	STAP1
FAIM3			WNT2	IRF4	CD37	GPR15	KLRB1	TBX21
				KIAA0125	CD3E	GPR171	LCK	TNFRSF4
				PNOC	CD3G	GPR183	LEPROTL1	TNFSF8
				RGS1	CD5	GPSM3	LTA	TRAF3IP3

	TNFRSF17	CD6	GZMA	LTB	TRAT1
	ZBP1	CD69	GZMB	LY9	ZAP70
		CD7	GZMK	NECAP2	

The gene sets referenced in this supplementary table are from Jiménez-Sánchez et al, 2019 (5). The gene sets in this table were generated using the R package Consensus TME (v.0.01.9000). Liver hepatocellular carcinoma was selected as the indication.

TME, tumor microenvironment**.**

Table S8. List of the genes that comprise the gene expression signature for the Bagaev immune set.

Angiogenesis	Checkpoint molecules	Effector cell traffic	Endothelium	Matrix remodeling	MHCI	Myeloid cells traffic	Th2	Treg and Th2 traffic	Treg	Tumor proliferation rate
VEGFA	PDCD1	CXCL9	NOS3	CA9	HLA-A	CSF2	IL4	CCL17	FOXP3	MKI67
VEGFB	CD274	CXCL10	KDR	MMP9	HLA-B	CSF3	IL5	CCL22	CTLA4	ESCO2
VEGFC	CTLA4	CXCL11	FLT1	MMP2	HLA-C	CXCL12	IL13	CCL1	IL10	CETN3
PDGFC	LAG3	CX3CL1	VCAM1	MMP1	B2M	CCL26	IL10	CCL28	TNFRSF18	CDK2
CXCL8	PDCD1LG2	CCL3	VWF	MMP3	TAP1	IL6	GATA3	CCR4	CCR8	CCND1
CXCR2	BTLA	CCL4	CDH5	MMP12	TAP2	CXCL8	CCR4	CCR8	IKZF4	CCNE1
FLT1	HAVCR2	CX3CR1	MMRN1	MMP7	TAPBP	CXCL5		CCR10	IKZF2	AURKA
PGF	TIGIT	CCL5	ENG	MMP11		CSF1R				AURKB
CXCL5	VSIR	CXCR3	CLEC14A	PLOD2		CSF2RA				E2F1

KDR	C10orf54	MMRN2	ADAMTS4	CSF3R	MYBL2
ANGPT1			ADAMTS5	CXCR4	BUB1
ANGPT2			LOX	IL6R	PLK1
TEK				CXCR2	CCNB1
VWF				CCL15	MCM2
CDH5				CSF1	MCM6

The gene sets referenced in this supplementary table are from Bagaev et al, 2021 (6).

REFERENCES

1. Petrick JL, Florio AA, Znaor A, Ruggieri D, Laversanne M, Alvarez CS, et al. International trends in hepatocellular carcinoma incidence, 1978-2012. Int J Cancer 2020;147:317-30.

2. Zhang X, El-Serag HB, Thrift AP. Sex and race disparities in the incidence of hepatocellular carcinoma in the United States examined through age-period-cohort analysis. Cancer Epidemiol Biomarkers Prev 2020;29:88-94.

3. Sung H, Ferlay J, Siegel RL, Laversanne M, Soerjomataram I, Jemal A, et al. Global cancer statistics 2020: GLOBOCAN estimates of incidence and mortality worldwide for 36 cancers in 185 countries. CA Cancer J Clin 2021;71:209-49.

4. Dasgupta P, Henshaw C, Youlden DR, Clark PJ, Aitken JF, Baade PD. Global trends in incidence rates of primary adult liver cancers: a systematic review and meta-analysis. Front Oncol 2020;10:171.

5. Jiménez-Sánchez A, Cast O, Miller ML. Comprehensive benchmarking and integration of tumor microenvironment cell estimation methods. Cancer Res 2019;79:6238-46.

6. Bagaev A, Kotlov N, Nomie K, Svekolkin V, Gafurov A, Isaeva O, et al. Conserved pan-cancer microenvironment subtypes predict response to immunotherapy. Cancer Cell 2021;39:845-65.e7.
